# Active Learning Performance in Labeling Radiology Images Is 90% Effective

**DOI:** 10.3389/fradi.2021.748968

**Published:** 2021-11-30

**Authors:** Patrick Bangert, Hankyu Moon, Jae Oh Woo, Sima Didari, Heng Hao

**Affiliations:** Samsung SDSA, San Jose, CA, United States

**Keywords:** artificial intelligence, computer vision, annotation, labeling, active learning, object discovery

## Abstract

To train artificial intelligence (AI) systems on radiology images, an image labeling step is necessary. Labeling for radiology images usually involves a human radiologist manually drawing a (polygonal) shape onto the image and attaching a word to it. As datasets are typically large, this task is repetitive, time-consuming, error-prone, and expensive. The AI methodology of active learning (AL) can assist human labelers by continuously sorting the unlabeled images in order of information gain and thus getting the labeler always to label the most informative image next. We find that after about 10%, depending on the dataset, of the images in a realistic dataset are labeled, virtually all the information content has been learnt and the remaining images can be automatically labeled. These images can then be checked by the radiologist, which is far easier and faster to do. In this way, the entire dataset is labeled with much less human effort. We introduce AL in detail and expose the effectiveness using three real-life datasets. We contribute five distinct elements to the standard AL workflow creating an advanced methodology.

## Introduction to Labeling

Artificial intelligence (AI) is a promising technology to help physicians in their daily tasks of finding and diagnosing a variety of conditions (1). Particularly in the realm of processing radiological images, AI can help in various ways. First, AI can sort the normal images (absence of any condition) from those with a condition, thus allowing the physician to focus on the important images. Second, AI can pre-screen images to detect and localize conditions that the physician then only needs to confirm, thus allowing the physician to be more efficient and spend more time with the human patient. Overall, it can be said objectively that multiple sources of diagnostic information improve the diagnostic result (2) in the decision-making process of what to do with a particular patient (3, 4).

Even beyond healthcare, companies are struggling to implement and benefit from AI as shown by the fact that only 1 in 10 companies are achieving significant financial benefits from AI. Avoiding three mistakes enhances the chances for success manyfold: (1) Ensure that the data is AI-ready, (2) initially deploy AI in a use case that has a well-defined return-on-investment as opposed to a toy problem, (3) and ensure that the team has all the necessary domain and AI expertise (5). This paper addresses the first of the challenges in the realm of radiology. Due to reducing the cost to get started with AI, this paper implicitly also addresses the second challenge.

Making an AI model requires training data, which includes both the input data that will be available in the practical application as well as the desired output data that that model is supposed to deliver. The desired output data is often called a “label” or “annotation.” In some cases, the label is measured by sensor equipment but in many cases, including most cases involving images, the label must be provided by a human domain expert. For all of AI, but especially in medical imaging, labeling the image data is the bottleneck of the field in the sense that it costs the most human labor by far (6).

**Figure 1:**
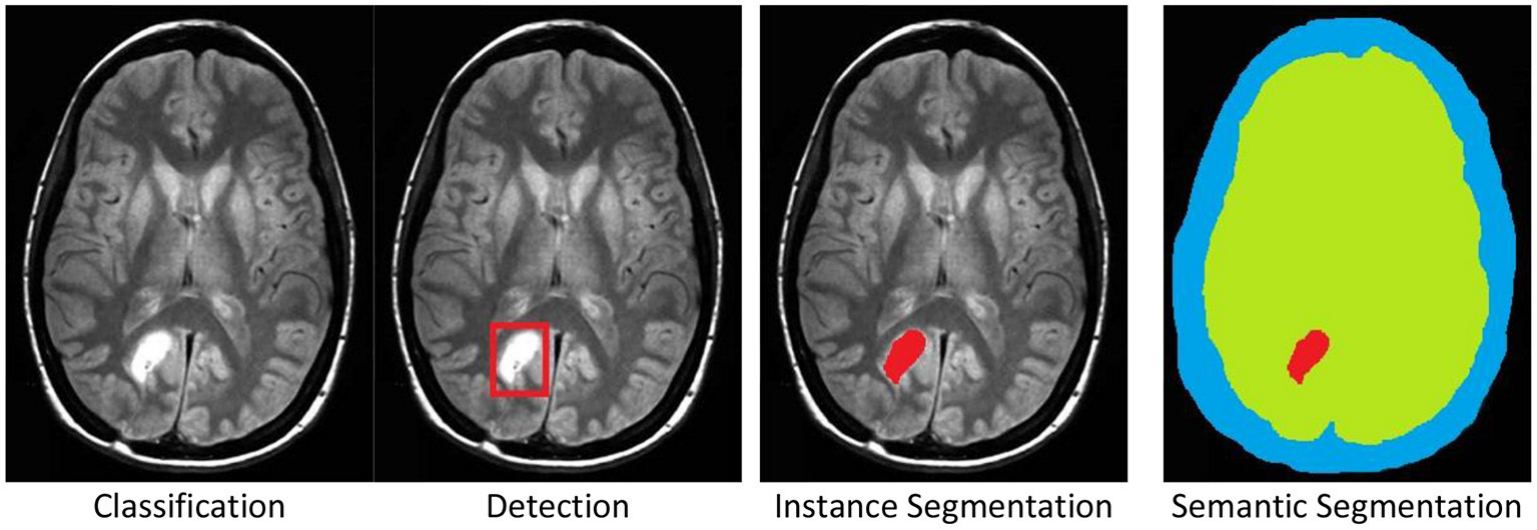
The four standard labeling cases illustrated by a brain tumor example in an MRI scan. Original image was published in Tamije Selvy et al. (7).

There are four standard labeling cases, (see Figure 1) for examples:

1. **Classification**: The entire image is associated to a category, e.g., tumor.
2. **Detection**: A part, or parts, of the image are marked by a rectangle, which is then associated to a category.
3. **Instance Segmentation**: A part, or parts, of the image are marked by a polygonal boundary, which is then associated to a category. If there are multiple instances of a single category, these instances are marked separately and distinguished.
4. **Semantic Segmentation**: All parts of the image are marked by polygonal boundaries, which are then associated to a category. If there are multiple instances of a single category, these are not marked separately and not distinguished.

Beyond these standard cases, there are a multitude of more specialized labeling cases for special-purpose AI models. While we will not discuss them here, the methods discussed below generalize to meet all labeling needs.

**Figure 2:**
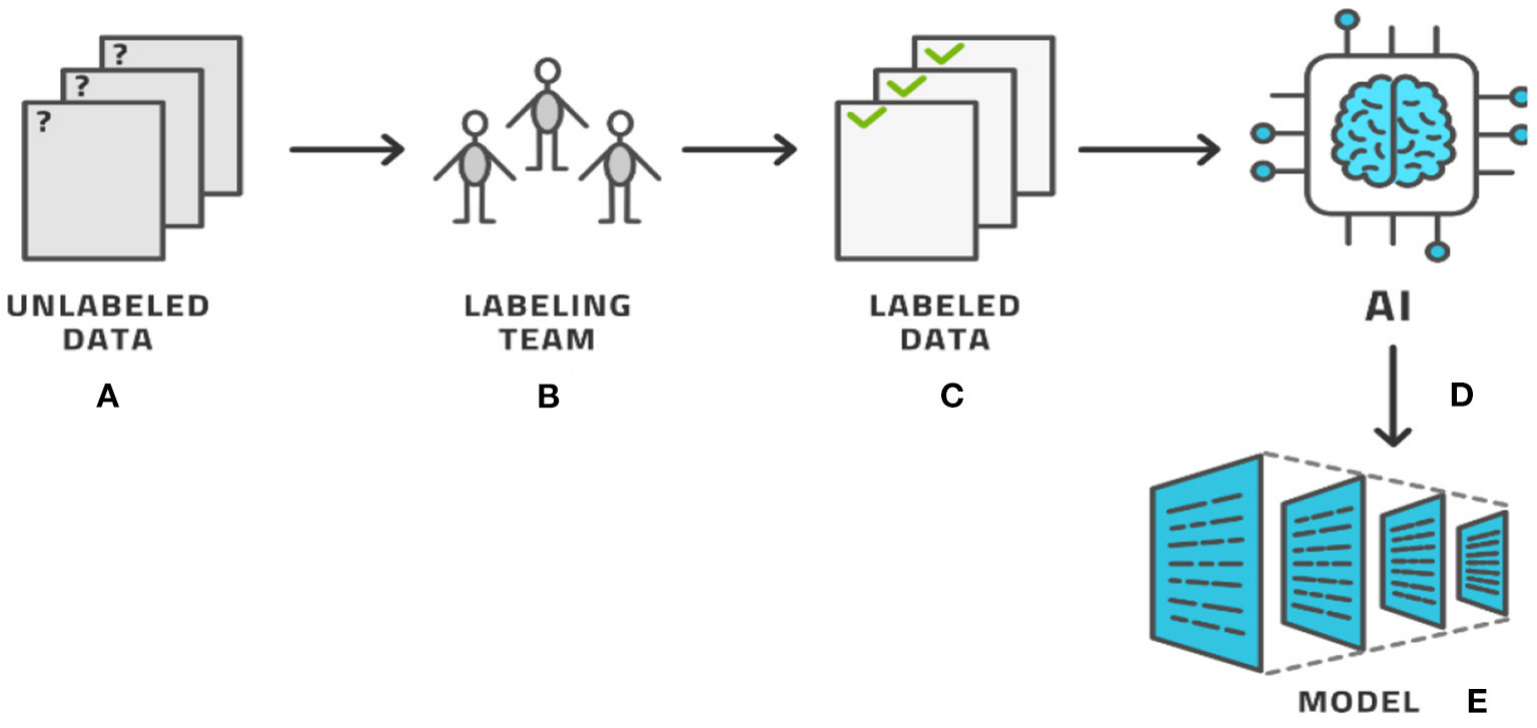
The basic AI workflow process where a dataset is labeled by experts and then used by AI methods to produce a model.

The process of labeling, as seen by these examples, typically involves a human expert clicking through various options to select the category and, in most cases, manually drawing on top of the image. The drawing must be accurate and so this activity consumes time. Providing a single image with segmentation labeling can easily require 2–15 min in simple cases and longer for more complex cases, per image and per person (8–11). To avoid human bias, each image is typically labeled by several human labelers with three labelers being a typical number (12, 13). For radiological use cases, the labeler must be a trained radiologist, who makes this process costly and temporarily prevents the radiologist from working with patients. Having labeled the entire dataset, AI methods produce the AI model that can then be used in clinical applications, (see Figure 2) for the basic AI workflow process. In this basic workflow, a vast literature exists on the last two steps—the training of the model and the models themselves. In practicality, the labeling process consumes most of the resources (12).

Typical Open-Data radiology datasets have several thousand to tens of thousands of images (14, 15) while proprietary datasets are often 10 times as large. For reference, a dataset of 100,000 images that are labeled by three labelers each using 10 min for each image will require a total of 24 person-years to label, considering that 1 month has 21 working days that have eight working hours. It would require a labeling team of 24 people to do this project in 1 year. Clearly, this is a level of effort and expense that represents a significant barrier to entry for any organization and makes many possible models commercially unviable. It is important to note that the training, testing, deploying, and maintaining of the AI model itself has not even begun at this point; this concerns only establishing the dataset that will form the basis for training.

This paper will present a methodology, known as active learning (AL), with which such datasets can be labeled with much less total human effort while achieving the same result, i.e., a fully labeled and human curated dataset. The idea of AL is not new, but we enhance the standard method in five important and novel ways (see section Methodology: Advanced Active Learning) and demonstrate their efficacy in three medical use cases.

In section Information and Active Learning, we review the basic ideas of AL. Section Methodology: Advanced Active Learning presents the five ways in which the basic ideas may be enhanced. Then, we present some results from practical case studies in section Results: Case Studies, discuss the place of AL in the larger ML Operations workflow in section Practical Use: Machine Learning Operations, and present the marketplace considerations for the technique in section Marketplace Considerations. Finally, we conclude and explain contrastive loss, which is an important concept for two of the five enhancements.

## Information and Active Learning

It is often implicitly assumed that the amount of data is proportional to the amount of information, or that each data point represents the same amount of information. There is a law of diminishing returns however, according to which some images deliver much information and others contribute only marginal added value (16–18).

While each image possesses a different inherent information value, each image represents approximately the same amount of human work to label it. If we could determine the most informative images and label only them, we might be able to train an intermediate AI model that would help us label the remaining images. We note in passing that while creating a (segmentation) label takes minutes, checking an already existing label for correctness only takes 10–20 s (9–11). If most of the labels proposed by this intermediate AI model are correct, the human workforce would be able to check these automatically-labeled images at a much faster rate than labeling them directly.

If we imagine that labeling takes minutes and reviewing takes seconds, the critical element in this process is how many images must be labeled manually for the automatic labeling to be accurate enough to deliver this quick reviewing process. Sticking to the above example of 100,000 images that take 10 min each to label for three labelers, if we needed to label only 10% of them and could review the other 90% at 20 s each for three reviewers, then we would consume a total of 3.2 years as opposed to 24 years, a saving of 87%. If the review is conducted by only one reviewer per image, we can reduce it by a further 0.5 years to achieve a saving of 89%. We thus find that the percentage of images that must be labeled and the percentage of human time and cost are approximately the same. This is the basis for our claim, in the title, that AL is 90% effective.

**Figure 3:**
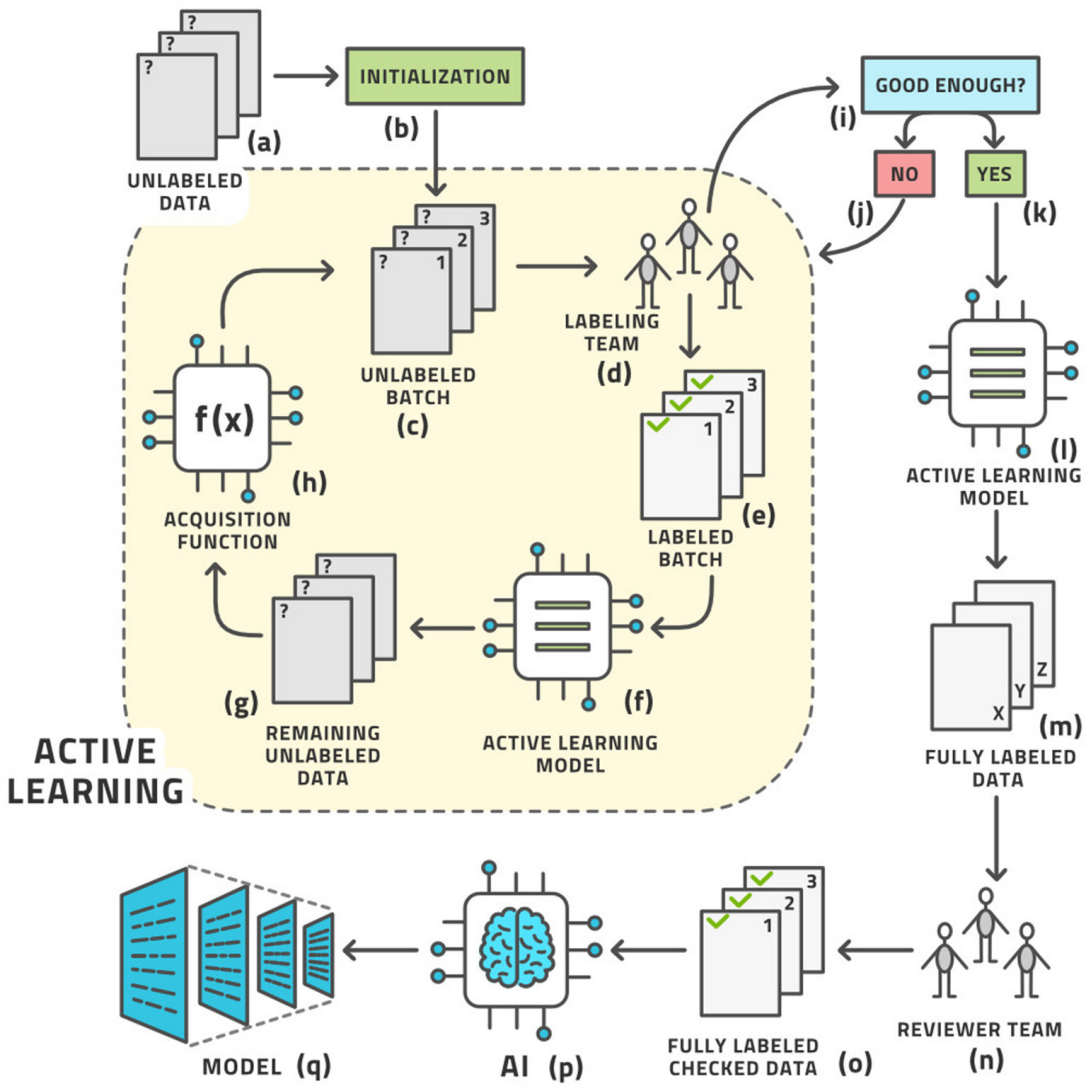
The standard active learning workflow.

As we cannot know the structure of phase space before venturing into it, we cannot determine the right images *a priori*. These ideas give rise to the concept of AL, which is the following process (19); (see Figure 3) for an illustration:

a. Start with a dataset of unlabeled images.
b. Initialize the process by (usually randomly) choosing a small number of images known as a batch from the pool of all unlabeled images. Practically, the batch size is the number of images that the labeling team can realistically label in 1 day.
c. We have the current batch of images to label.
d. Let the labeling team label these images.
e. The current batch is now labeled.
f. An AI model, known as the AL model, is now trained on (most of) the labeled images and is capable of automatically labeling any similar image with a certain confidence in its own accuracy. The accuracy is tracked by retaining some labeled images for testing this model.
g. This model is applied to the remaining unlabeled data.
h. If the accuracy is high enough, it becomes economical to stop the loop.
i. The AL model is then applied to all unlabeled data.
j. This application results in the entire dataset having labels; some by the human team and some by the AL model.
k. As many labels are provided by the model that is known to have good (but not 100%) accuracy, the auto-labels must be reviewed or edited by a reviewer team that is almost always the same as the labeling team.
l. After review, the entire dataset has labels that were either produced by human experts or approved/edited by them.
m. Artificial intelligence modeling on the fully labeled dataset can now be started.
n. The diagnostic model for clinical use is the result.

This process involves a crucial decision: When is the accuracy high enough to stop manual labeling and proceed to reviewing automatically-produced labels? Generally the accuracy increases sharply at the start of the process and starts to level off after some time. Finding this “elbow point” as the curve evolves is one element in this decision. The other element is the inherent accuracy requirement of the task at hand and the amount of human labor required to correct a sub-optimal label. In most practical cases 90–95% accuracy is fine while reminding the reader again that this is only the intermediate model meant to help with labeling and not the final diagnostic model intended for clinical use. We shall see below that some datasets allow such accuracy to be achieved readily while for others it remains aspirational and we must be contented with around 85% only.

**Figure 4:**
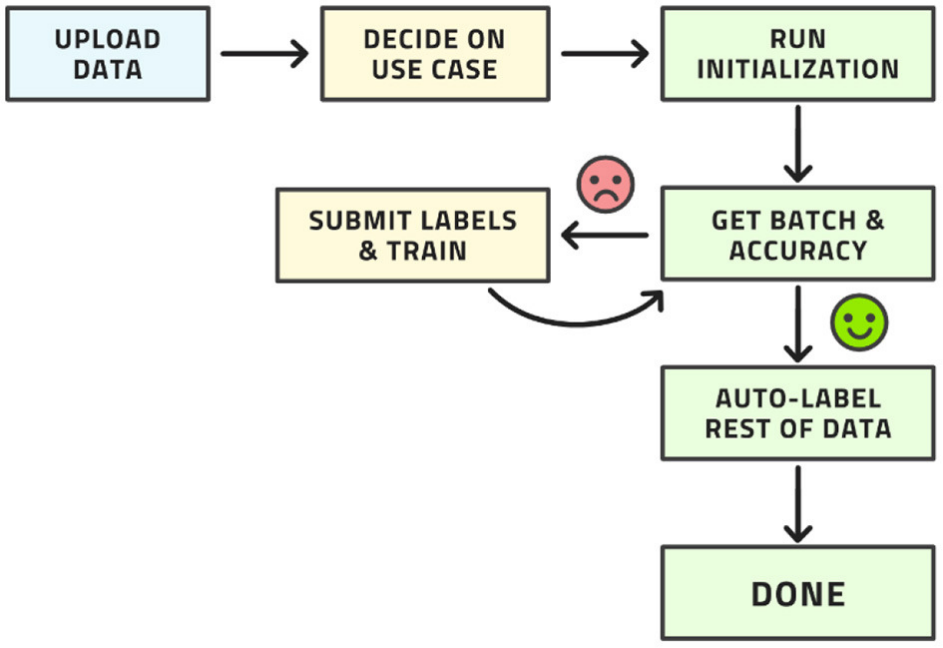
Workflow from the point of view of the labeling team.

This workflow may look complex, but all its steps can be readily automated. The labeling team does not need to act any differently than before. They are served images for labeling, which they then do. The difference is that the order of the images to be labeled is carefully curated by AL whereas the order was usually random before. The process from the point of view of the labeling is shown in Figure 4.

Active learning was first suggested in 1996 (19) and is a well-known idea and methodology in research circles today. Its applicability in commercial software and its practical use, particularly in medical applications, is limited. The method was thoroughly studied in generality (20–22). In particular Bayesian approaches were added later (23) and various applications to medical image classification studied (24). These studies lead to what we call standard AL in this paper.

The workflow described above can be called standard AL. It is possible to make this procedure better in the sense of making it converge to a certain accuracy after fewer iterations or after fewer manually labeled images. In the following section, we describe our new AL methodology with five novel elements that enhance standard AL. These elements represent the scientific contribution of this paper.

## Methodology: Advanced Active Learning

**Figure 5:**
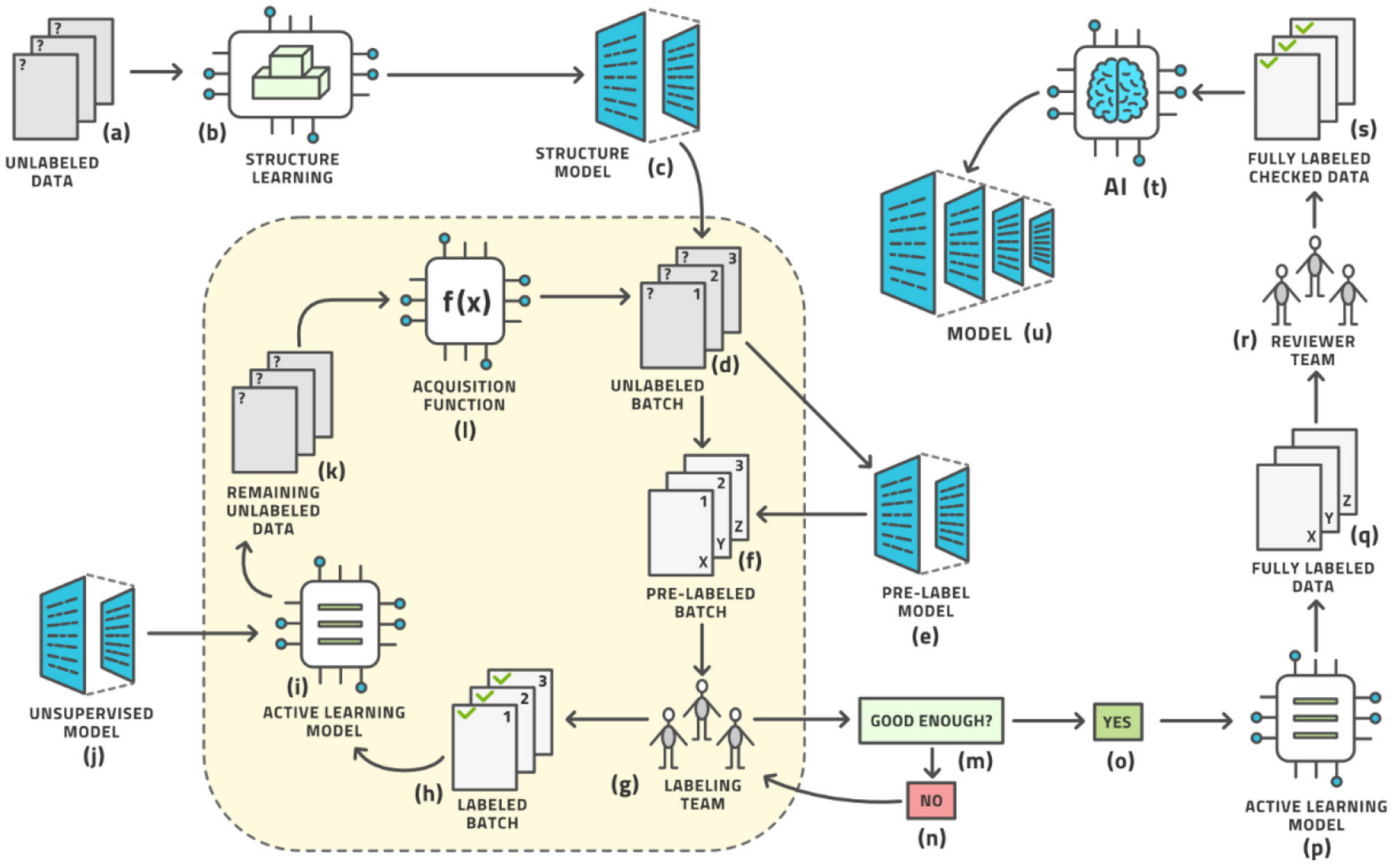
The advanced active learning workflow (compare with Figure 3).

The standard workflow can be enhanced in five places to further reduce the amount of human labor until the system can auto-label the remaining images with relatively high accuracy. Our advanced AL workflow is displayed in Figure 5.

Each of the five techniques introduced here is described in detail in other publications (several of which are written by the same authors), which are cited here. This paper discusses them all in an overarching context of the entire AL workflow and in concert with each other. In section Results: Case Studies, we demonstrate some results when all five techniques are used at the same time, as opposed to only one at a time, which is common in the cited specialized technical publications.

In this sense, the architecture diagram of Figure 5 is the real novelty of this paper and this is the subject of this section, which briefly outlines each aspect and provides references for more detailed study.

First, the initialization of the AL loop does not have to be random but can be made based on unsupervised techniques (steps b and c in Figure 5), as no labels exist at this point. We attempt to learn the structure of the distribution of images and represent this as a structure model that clusters images based on similarity. The SimCLR framework has proven to be particularly effective in this regard (25, 26), which optimizes for contrastive loss, see Appendix for an explanation (27, 28). This unsupervised initializer turns the space of images into a vector space of latent vectors, the distances between which allow the generation of clusters. The data points for the first batch to be labeled are then chosen from a distribution over these clusters to allow for maximal diversity of information at the start of the process (29).

Second, a pre-labeling model (steps e and f in Figure 5) can be added before the human labeling team provides its input. This model supplies an (approximate) label so that the human team merely corrects a reasonable first guess at a label thereby reducing the labor time. Any such model would have had to be trained previously on some other dataset; the more similar this prior dataset is to the one under study, the better the pre-labeling model will be able to initialize the label. This leads into the idea of reproducible research or model lifecycle management: After a model is trained on a dataset, some research team may acquire a second dataset for the same situation and wish to label it using the AL loop described here. The initial model would be the pre-label model for the second dataset. The pre-label step is the AL version of the general concept of bootstrapping.

Third, the AL model itself must be chosen well for the task. Numerous models have been investigated (20, 24). We have chosen the Gaussian Process (30), on top of the SimCLR foundation mentioned above, and observe superior results (29). We postulate that this method is less affected by an imbalance in the dataset than prior methods, which is a very desirable property in radiology as the “normal” condition will have many more examples than various non-normal conditions, and some conditions are rarer than others. In fact, it seems unrealistic to expect a balanced dataset under real-life conditions anywhere in healthcare.

**Figure 6:**
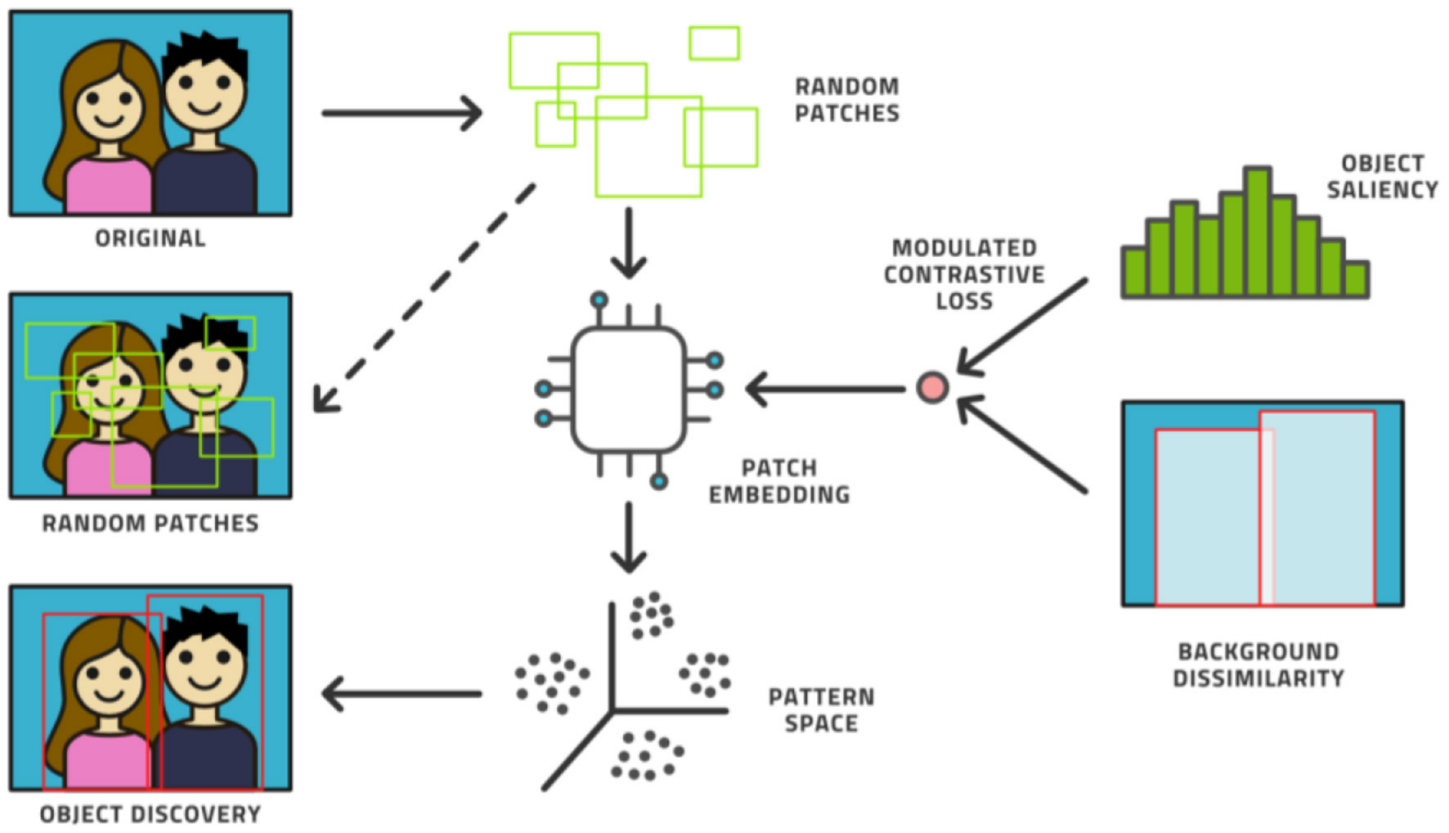
The unsupervised object discovery process.

Fourth, the AL model can be augmented with an unsupervised model. Novel ways have been found to support object detection in an unsupervised way (31–33) from a small number of images (200 or less) in which certain objects appear frequently against simple backgrounds. This process randomly chooses many rectangular patches from each image and then trains an embedding into pattern space by contrastive training, (see Figure 6). Objects are distinguished from background patterns by modulating the contrastive loss based on two assumptions: (1) objects are relatively small and surrounded by background, and common background patterns are relatively larger and more uniform across images, and (2) the color distribution inside versus outside the bounding box of an object is expected to be distinct in a statistically significant manner. Such models could be trained on the initial dataset without human effort being expended and then assist the labeling effort by localizing the areas in need of attention (34).

**Figure 7:**
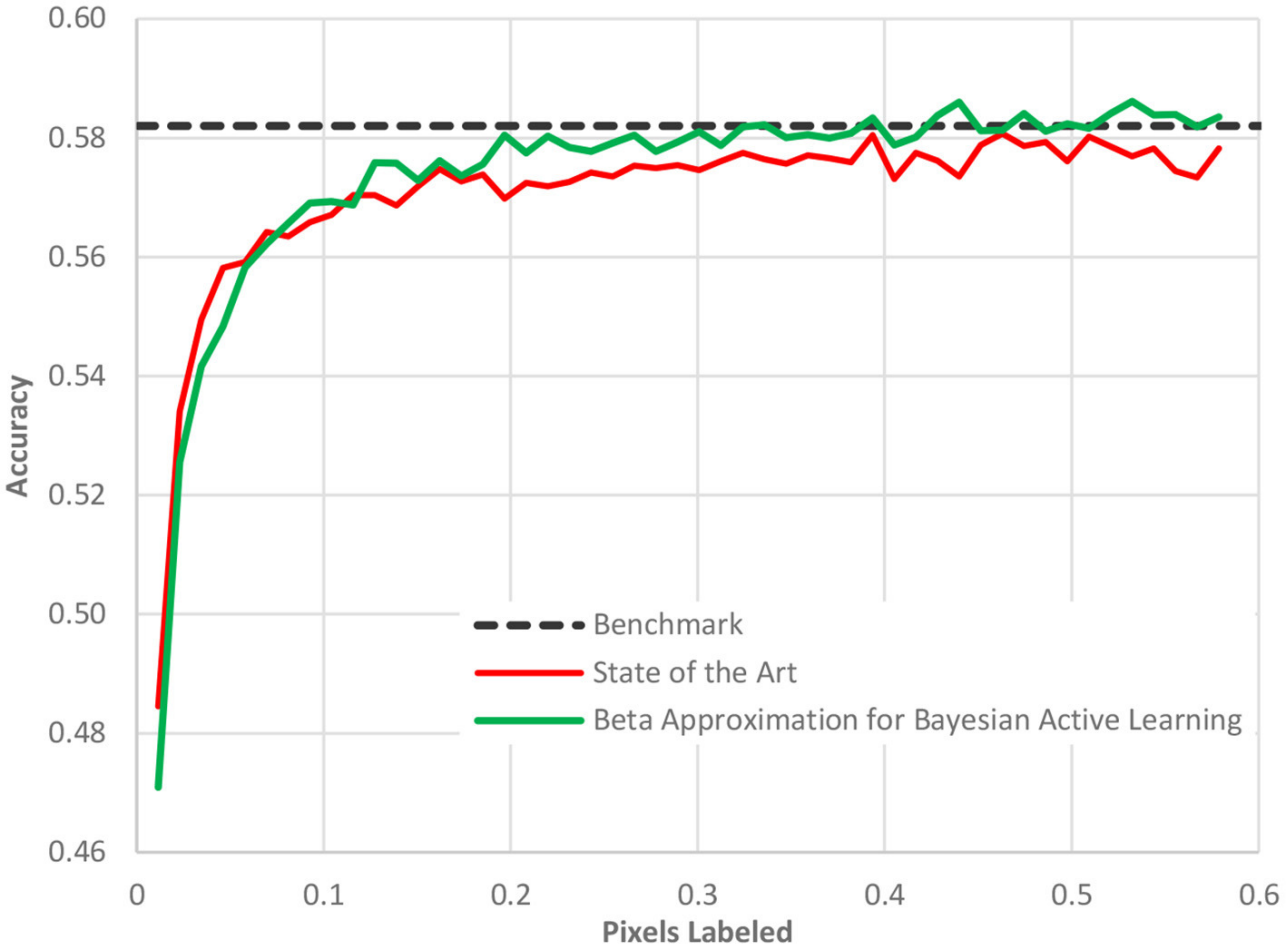
The performance of our acquisition function, Beta approximation for Bayesian active learning, relative to the state of the art active learning method and a benchmark accuracy based on a full AI model.

Fifth, the acquisition function can be made more sophisticated beyond the standard choices (35). An area of active research, a better acquisition function can better identify the most informative data points and sort them to the top of the ranking list. As the batch size is usually limited by organizational constraints, the ranking of images is crucial for the convergence of the entire workflow. Our new acquisition function, Beta approximation for Bayesian active learning (BABA), outperforms the state of the art by approximately 40% in terms of labeling effort required to get to a stable accuracy, [see Figure 7 (36)].

In Figure 7, as in all the case study figures to follow, the benefit must be read horizontally. The horizontal axis displays the amount of human labeling injected into the project. The vertical axis represents the AL model accuracy. We detect the benefit of the method by determining when (in terms of labeling) we reach a certain cut-off accuracy. We observe in Figure 7 that the benchmark accuracy is reached using AL already after only 20% of the dataset is labeled while the state-of-the-art AL framework requires nearly 40% of the dataset to be labeled. The difference is entirely due to the new acquisition function as all other elements of the process were identical.

## Results: Case Studies

We will examine this approach in three different scenarios. First, we will look at lung x-ray images to detect Covid-19. Second, we look at microscopic slide images to detect breast cancer. Third, we will look at colonoscopy videos to detect the cleanliness of the colon. In all three examples, the datasets are available publicly and are expertly annotated so that the ground truth is known.

**Figure 8:**
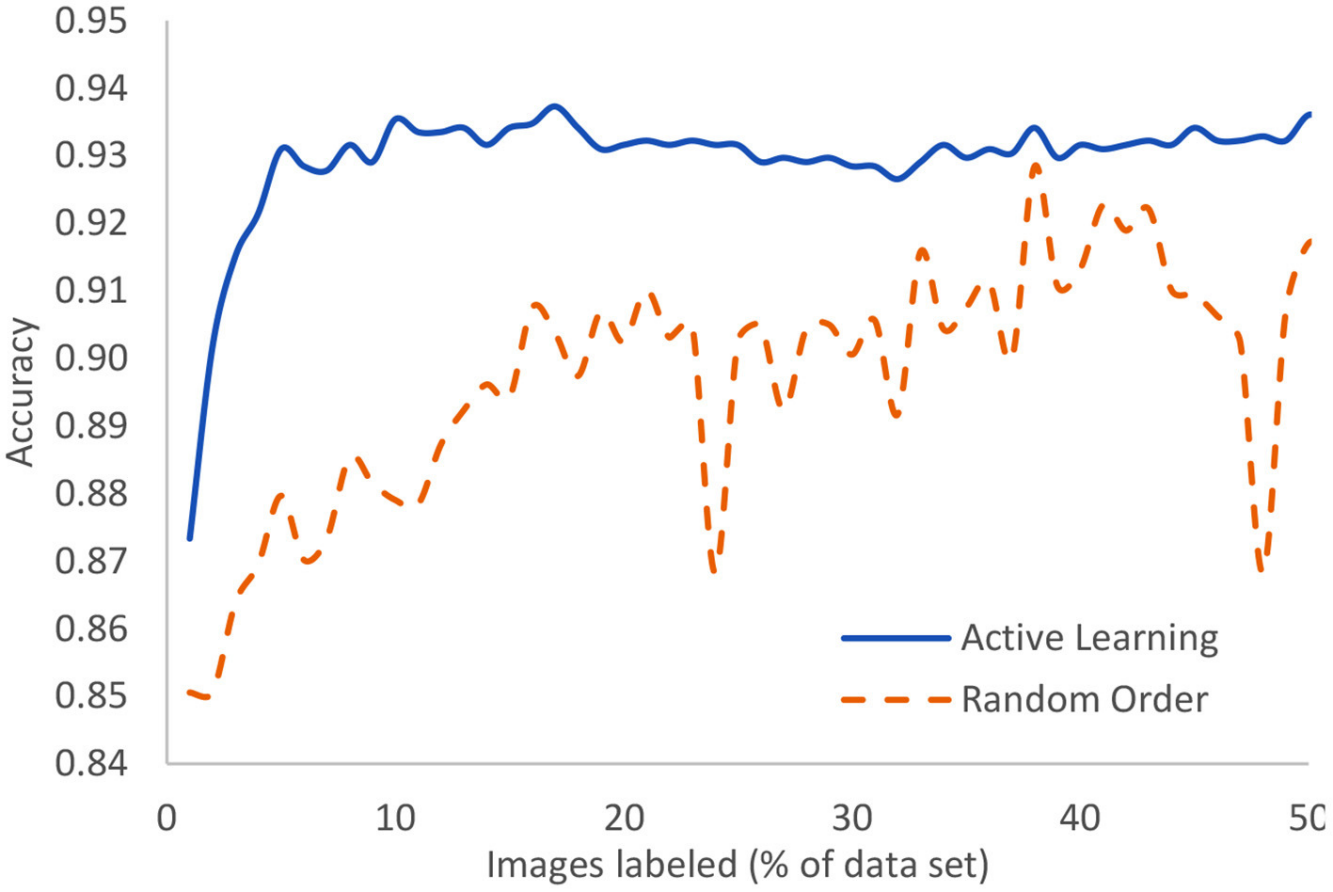
In the case of Covid-19 detection from lung X-rays, this displays the growth of accuracy due to active learning vs. random order labeling.

The Covid-19 dataset consists of 15,521 chest x-ray images of which 8,851 are normal, 6,063 are pneumonia, and 601 are Covid-19 examples. It is an assemblage of five open datasets (37–41). The task is a classification task in which the entire image is to be associated to one of three classes: normal, pneumonia, or Covid-19. These were processed using our novel AL method with the result that after only 5% of the images were human-labeled, the model had achieved 93.1% accuracy. The highest currently-achievable accuracy on this dataset is 93.7% once the entire dataset is labeled. Standard active learning requires about 16% of the data to be human-labeled in order to achieve comparable accuracy to our novel method. We clearly see the diminishing returns on the human labor of labeling. Figure 8 displays the evolution of accuracy as the labeling team labels the dataset 1% at a time both using active learning (blue, solid) and using the normal random order (orange, dashed) (29).

**Figure 9:**
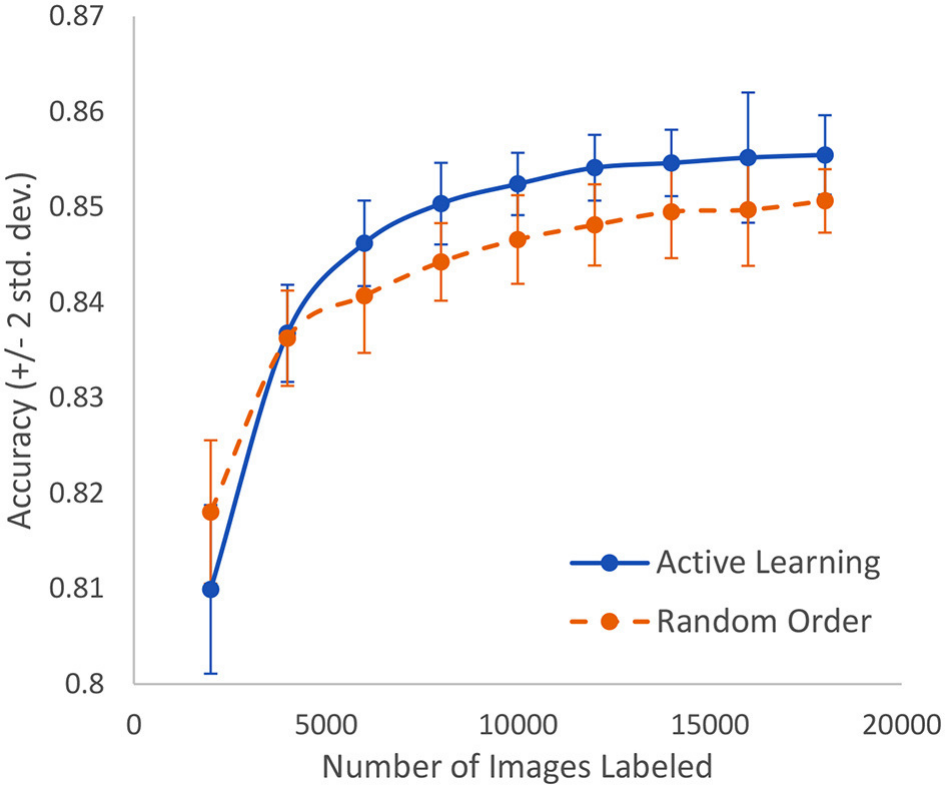
In the case of breast cancer detection based on microscopic slide images, this represents the growth of accuracy due to active learning vs. random order labeling. Both curves have error bars that are equal to two standard deviations about the mean.

A similar study is made using microscopic slide images to detect breast cancer at the cellular level. The dataset includes 50,286 images that are fully expertly annotated (42). The task is to classify images into one of two categories: normal or mitosis. This is a very difficult problem where the best known model achieves an accuracy of 88%. As shown in Figure 9, we can achieve 85% accuracy after labeling only 16% of the data as compared to having to label 36% of the data in a random order to achieve the same accuracy. In other words, we can lower the human labor by 56% in this case. Standard active learning would require about 25% to achieve similar accuracy.

**Figure 10:**
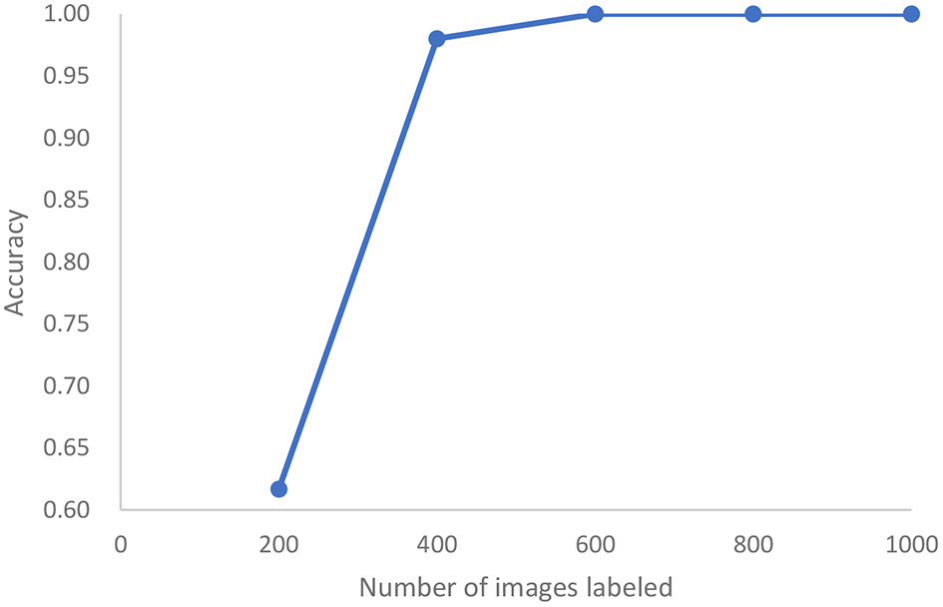
In the case of colonoscopy detection, this represents the growth of accuracy of active learning as a function of the number of images that are labeled.

In a third study, we analyze the cleanliness of the colon as described in a dataset of 5,525 images extracted from 21 colonoscopy videos (43). This is also an image classification problem with five classes of cleanliness. We find that after labeling only 400 of these images, we can achieve 98% accuracy and reach 100% accuracy after 600 images are labeled, (see Figure 10). Standard active learning would require 1,000 images to be labeled. For the purposes of active learning, we thus achieve an effort reduction of 93%.

As all of these case studies are based on image classification, the results are comparable. The differences in the required amount of human labeling must be interpreted to be due to the difference in problem difficulty. The active learning model included here is generic in that it can be used for any image classification task and has in no way been tuned to these particular cases. In particular, it was the same model and same active learning methodology for all cases.

Depending on the complexity of the task and the diversity of the data, we conclude that active learning can save between 56 and 93% of the human labor required to label a dataset in preparation to AI modeling.

## Practical Use: Machine Learning Operations

**Figure 11:**
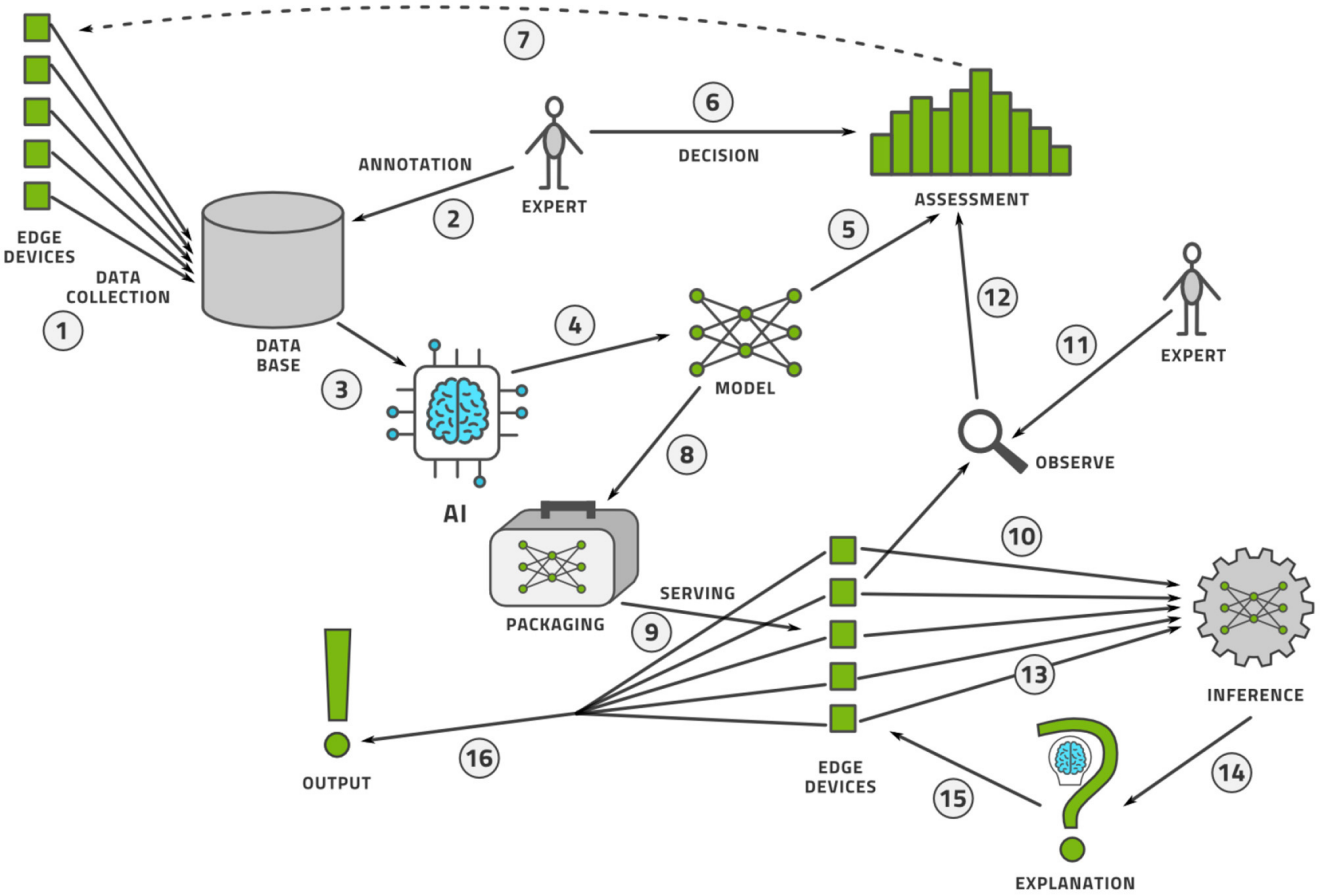
The MLOps lifecycle management flow for an AI model.

Once the data is labeled, the AI model can be trained and deployed for practical use. The process does not stop here however. Like software, AI models are never finished. The life-cycle management procedure for AI models is known as machine learning operations, or MLOps. We illustrate this process in Figure 11 and describe it here.

1. Data is collected from a fleet of edge devices and stored in some central location. In medical imaging, the edge devices might be MRI scanners, CT scanners, ultrasound machines, and so on.
2. Human experts annotate this data, perhaps using active learning as illustrated above.
3. The data is provided to an AI modeling suite that performs feature engineering, model selection, and hyper-parameter tuning (44). The modeling procedure usually takes place on powerful computers in a data center.
4. The outcome of the AI modeling process is the model itself.
5. The model must be assessed for accuracy (bias), variance, and robustness.
6. Having made an objective assessment, human experts must make a subjective decision to accept or reject the model.
7. If the model is not good enough, perhaps more data must be collected and this closes the model creation loop.
8. If the model is good enough, it must be packaged to run on an edge device. This generally implies (a) model compression so that the model fits into the memory and computational infrastructure requirements of the edge device and (b) provision of a run-time environment that executes the model and provides the output.
9. The packaged model is then served, which implies the transference onto a fleet of edge devices alongside the record-keeping of which version of the model was deployed where.
10. As the model is used at the edge, some data points are specially selected for observation.
11. Every observed data point must be annotated by a human expert to see if the model output agrees with the human annotation.
12. These observability data flow into a continual assessment that may, or may not, trigger a re-training of the model in the model creation loop.
13. Every data point at the edge is provided to the run-time environment, which executes the model on the data.
14. The model output may then need to be provided to an explainability engine, which produces a type of output that may help the user of the edge device understand how the model produced its output. Preferably, the model itself can provide interpretable output.
15. The edge device receives this explanation and the model output.
16. Finally, the output of model and explanation are provided to the user in an understandable way.

We see that MLOps consists of two interconnected loops that make a model and continuously observe its performance in the wild. In this way, we can assure that the model is always performing up to the highest scientific, transparency, and ethical standards.

## Marketplace Considerations

There are high commercial expectations of AI in healthcare to diagnose, manage, and treat a variety of conditions (45), the value of which is projected to be US$28B by 2025 (46). Using AI “we can innovate procedures to be more productive with better outcomes,” according to Frans van Houten, CEO of Philips (47). The Food and Drug Administration of the USA government (FDA) has published a discussion paper in 2019 followed by a goals paper in 2021 (48) that should culminate in a policy paper in the future governing the approval and regulation of Software as a Medical Device (SaMD), which includes AI-based models. There are five goals in this plan: (1) a regulatory framework, (2) best practices for AI modeling, (3) transparency to patients, (4) methods to evaluate and address bias and robustness, and (5) monitoring real-world performance of AI models. It is our interpretation of these goals that transparency may be interpreted as the AI topics of explainability and interpretability; bias and robustness go beyond the usual numerical performance measures and include AI Ethics, while the real-world performance is usually referred to as MLOps.

There are 64 FDA approved AI-based medical devices as of 2020, mostly in the areas of radiology and cardiology, where the definition of AI however remains unclear (49). Due to the uncertain definition of AI, the count of such devices reached 130 in 2021 (50). Platforms exist that allow the commercial sharing of AI models intended for medical use in, for example, the IntelliSpace AI Workflow Suite by Philips (51) and Edison Open AI Orchestrator by General Electric Healthcare (52).

Obstacles for adopting AI in daily clinical practice include transparency, concerns about bias, explainability (53), and trust (54). Risks include the misuse of an AI model intended for decision support as a primary diagnostic tool (50). If the training data of an AI model is not representative of the population, it may see quite different accuracy ratings on real-life datasets (50), which is a significant risk for health care in which it is difficult and expensive to acquire suitably diverse training data. Diversity in this sense does not only refer to diversity of human subjects but also diversity of device technologies, photography angles, image resolutions, camera focus settings, and so on.

Processing medical information with AI systems requires thought about explainability and ethics as well as legal challenges (55) with some early frameworks being proposed (56). Processing medical information in the cloud poses additional challenges that must be considered (57). Anonymizing medical data is often a legal requirement depending on the software and hardware architecture and must be designed carefully (58). Whenever AI is applied to people's lives, it should be ethical and fair. What those terms mean however, is controversial as AI methods—being mathematical—require significantly more precise definitions of these terms than human beings usually desire. For example, a non-exhaustive list of 21 distinct definitions of fairness was compiled (59). It can also be demonstrated that any one model cannot adhere to multiple definitions of fairness simultaneously except under specific, rare conditions (60, 61). Ultimately, this is a multi-criterion optimization problem that almost always requires a compromise between the different desiderata (62).

Current human culture expects humans to be fallible and accepts a certain error-rate in its human experts. This is not true for human-made systems, which are expected to have error-rates that are several orders of magnitude lower (63). For example, in the early days of AI, an AI system that had demonstrably lower racial bias than the human process it was meant to replace was discontinued because it was not equitable enough (64). For this reason, it should be our aspirational goal as the AI community to develop inherently interpretable and explainable models (65) even though interpretability is not a universally definable notion (66–68). In fact, numerous circumstances exist where an inherently interpretable model has comparable accuracy to a black-box model and is thus far more valuable in practice (65, 69). Even for computer vision, interpretable models can be constructed. For example, the model may point to certain parts of an image that look like an example image from the training data (70). The approach “this looks like that” is a frequent method of explanation by human domain experts where images are concerned.

## Conclusion

We conclude that the human effort required to label an image dataset can be reduced by approximately 90% in most cases by using the described advanced active learning workflow. In exceptionally difficult circumstances, this may be as low as 50–60%. In any case, the labor saving is measured in tens of person-years for a realistic project and so is highly significant. We have demonstrated the efficacy of five distinct and novel elements by which standard active learning is enhanced to deliver a significant additional reduction of labeling effort: (1) initializing the loop with an unsupervised clustering model, (2) adding a pre-labeling model, (3) using a standard SimCLR and Gaussian Process model as the centerpiece for active learning, (4) enhancing the central model with an unsupervised model, and (5) using the novel Bayesian acquisition function BABA.

As image labeling represents the bulk of the human effort required for computer vision AI, this enablement technology makes many previously commercially inaccessible use cases realistic. We hope to demonstrate this on many more specific use cases in the future.

## Data Availability Statement

The original data presented in the study are publicly available at the cited links. Further inquiries can be directed to the corresponding author.

## Author Contributions

PB wrote the paper and organized the case studies. HM supervised the research and did the object discovery. JW invented the new acquisition function. SD worked on segmentation. HH led the case studies. All authors were involved in the research and case studies.

## Conflict of Interest

All authors are employed by Samsung SDS America Inc. The authors declare that the research was conducted in the absence of any commercial or financial relationships that could be construed as a potential conflict of interest.

## Publisher's Note

All claims expressed in this article are solely those of the authors and do not necessarily represent those of their affiliated organizations, or those of the publisher, the editors and the reviewers. Any product that may be evaluated in this article, or claim that may be made by its manufacturer, is not guaranteed or endorsed by the publisher.
