# Supplementary material for: Active Learning Performance in Labeling Radiology Images Is 90% Effective
Source: Front Radiol. 2021 Nov 30;1:748968. doi: 10.3389/fradi.2021.748968 (PMC10365082; doi:10.3389/fradi.2021.748968)
Supplement: Supplementary file 1 [file Data_Sheet_1.docx]

# Appendix: Contrastive Loss

The concept of *contrastive loss* is central to many modern image-processing AI systems, and it is instructive to introduce it here since we have referred to it multiple times in the above paper. A *metric* is a function that measures the distance between two objects. In the physical world, we may use the Euclidean metric that measures the straight-line distance in miles between towns or millimeters from one end of a tumor to the other. Also useful is the Manhattan metric that measures the distance between two points in a designed city like Manhattan not in terms of the straight-line but rather in the number of city-blocks that must be walked between two points. For a pedestrian in Manhattan, the Manhattan metric is useful while the Euclidean metric is not (as the straight line between two points cannot practically be walked on). Therefore, for a particular task, we must select the correct metric.

When trying to identify certain images, or parts of images, as examples of either the same or a different underlying condition (including the “normal” condition), we could try to formulate this as a question about a metric. We first project the image into a vector space, i.e. we turn the image into a sequence of numbers somehow. Then, we compute the distance between any pair of such points, and that is the metric value between the images. For the purpose of contrastive learning, we use the cosine distance, i.e. the cosine of the angle between two vectors, as the metric.


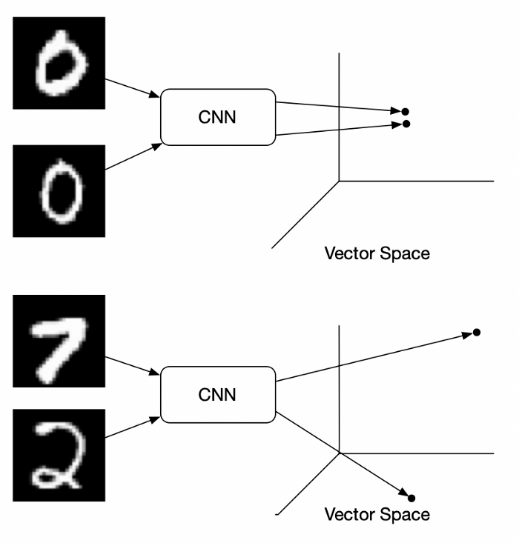


Figure 1. Contrastive loss explained for similar and dissimilar pairs of examples.

In our AI context, the method of projecting the image into a vector space is an AI model. Our expectation is that if we put two images into this model that are “similar” to each other in terms of the situation that we wish to model, then we expect the distance between them to be small. On the other hand, if the two images are “different” from each other, then the distance should be large. This general idea that similarity and distance are related is referred to as contrastive loss, see Figure 12. Note that we are *not* concerned with the *location* of each image in the vector space but only with the distance between them. The SimCLR method (Chen, 2020a) (Chen, 2020b) is an example of this idea where a specific implementation of an AI model is used to calculate such distances with great effect.
